# A Complex Case of Renal Artery Stenosis in a 3-Year-Old Patient with Neurofibromatosis Type 1 and Secondary Hypertension

**DOI:** 10.3390/diagnostics16071047

**Published:** 2026-03-31

**Authors:** Jakub Pytlos, Piotr Majcher, Piotr Skrzypczyk, Rafał Maciąg, Bożena Werner, Mariusz Furmanek

**Affiliations:** 1 Department of Pediatric Radiology, Medical University of Warsaw, 02-091 Warsaw, Poland; 2 2nd Department of Clinical Radiology, Doctoral School, Medical University of Warsaw, 02-091 Warsaw, Poland; 3 Department of Pediatrics and Nephrology, Medical University of Warsaw, 02-091 Warsaw, Poland; pskrzypczyk@wum.edu.pl; 4 2nd Department of Clinical Radiology, Medical University of Warsaw, 02-091 Warsaw, Poland; 5 Department of Pediatric Cardiology and General Pediatrics, Medical University of Warsaw, 02-091 Warsaw, Poland

**Keywords:** renal artery stenosis, vascular fistula, neurofibromatosis 1, hypertension, percutaneous transluminal angioplasty, Doppler ultrasonography, computed tomography angiography

## Abstract

We describe a case of a 3-year-old girl with neurofibromatosis type 1 presenting with arterial hypertension, in whom multimodal vascular imaging identified significant right renal artery stenosis. The patient was successfully treated with percutaneous transluminal renal angioplasty; however, post-procedural Doppler ultrasound revealed a transient vascular fistula. Changes in renal arterial inflow during the procedure may have temporarily altered pressure gradients, facilitating the opening of communication involving pre-existing compensatory collateral vessels. This case illustrates the diagnostic value of multimodal vascular imaging in pediatric hypertension and highlights a rare, self-limiting post-interventional vascular phenomenon.

**Figure 1:**
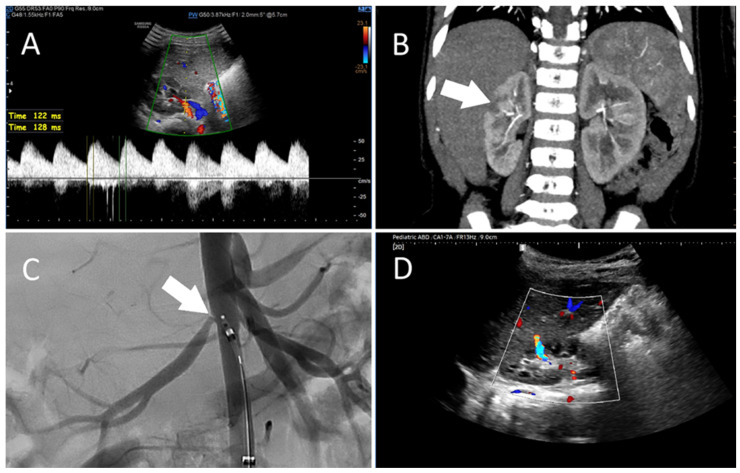
Imaging findings in a 3-year-old female patient with neurofibromatosis type 1 (NF1) and right renal artery stenosis. The patient with a known history of NF1 was referred to the Pediatric Nephrology Clinic due to arterial hypertension, which persisted despite treatment with amlodipine and ramipril and later with amlodipine and doxazosin. On admission, 24 h ambulatory blood pressure monitoring demonstrated systolic values exceeding the 99th percentile and diastolic values around the 95th percentile (a 24 h average of 119/72 mmHg, a daytime average of 126/78 mmHg, and a nighttime average of 112/65 mmHg). Laboratory tests showed an elevated albumin-to-creatinine ratio, hyperreninemia and secondary hyperaldosteronism. Echocardiography was unremarkable. **Doppler ultrasound** (**A**) demonstrated abnormal flow within the right renal artery and its segmental branches distal to the site of stenosis, with markedly prolonged acceleration times of 120–130 ms, as well as renal asymmetry (right: 63 mm; left: 75 mm). **Computed tomography angiography** (CTA, (**B**)) confirmed significant stenosis of the right renal artery with post-stenotic dilatation and right renal hypotrophy, as well as recognized areas of reduced contrast enhancement in the cortical layer of the upper and middle poles (arrow), consistent with an area of cortical hypoperfusion. The left renal artery was unaffected. Subsequently, a percutaneous transluminal renal angioplasty (PTRA) was performed, with intraprocedural **digital subtraction angiography** (**C**) demonstrating a focal ostial stenosis of the right renal artery (arrow), which was successfully dilated. On follow-up, **Doppler ultrasound** (**D**) demonstrated a narrow vessel with turbulent flow (~130 cm/s) originating in the upper pole of the kidney, carrying blood from the outer margin of the kidney toward the renal hilum, consistent with a vascular fistula. A repeat Doppler ultrasound procedure performed five days later did not reveal the previously identified unusual renal blood flow pattern. Flow parameters, including acceleration time, in the right renal artery and its segmental branches were within normal limits. NF1 is a recognized cause of hypertension due to renal artery stenosis [1]. The narrowing observed reduces renal perfusion, triggering activation of the renin–angiotensin–aldosterone (RAA) system, as well as suppression of renin secretion and pressure natriuresis from the unaffected side. The resulting chronic ischemia may also promote the development of collateral vessels as an adaptive response in order to maintain adequate blood flow within the kidney [2]. Over time, these compensatory vessels may become increasingly prominent, helping to partially maintain perfusion despite ongoing arterial stenosis. In our patient, the fistula likely developed in the context of compensatory collateral vessel formation, consistent with the earlier CTA findings of reduced cortical perfusion in the upper and middle poles. The sudden hemodynamic shift following changes in renal arterial inflow during PTRA may have transiently altered pressure gradients, facilitating the opening of communication between collateral vessels and the renal hilum. Once formed, the restored arterial flow would preferentially follow the path of least resistance, temporarily sustaining the shunt. Fistulas of this type are generally self-limiting and tend to close following adequate treatment as improved perfusion reduces abnormal pressure gradients [3,4]. A similar outcome was observed in our patient. Achieving effective blood pressure control was crucial for clinical improvement of this patient. This highlights the need to consider renal artery stenosis as a potential underlying cause in similar cases. Timely recognition and targeted management combining pharmacological and endovascular therapy is essential for achieving favorable outcomes.

## Data Availability

The original contributions presented in this study are included in the article. Further inquiries can be directed to the corresponding author.

## Abbreviations

The following abbreviations are used in this manuscript:

NF1	Neurofibromatosis type 1
CTA	Computed tomography angiography
PTRA	Percutaneous transluminal renal angioplasty
RAA	Renin–angiotensin–aldosterone (system)
